# Characterisation and In Vitro Drug Release Profiles of Oleanolic Acid- and Asiatic Acid-Loaded Solid Lipid Nanoparticles (SLNs) for Oral Administration

**DOI:** 10.3390/pharmaceutics17060723

**Published:** 2025-05-30

**Authors:** Michael Oboh, Eman Elhassan, Neil Anthony Koorbanally, Laurencia Govender, Muthulisi Siwela, Thirumala Govender, Blessing Nkazimulo Mkhwanazi

**Affiliations:** 1 Discipline of Dietetics and Human Nutrition, School of Agricultural, Earth and Environmental Sciences, College of Agriculture, Engineering and Science, University of KwaZulu-Natal, Pietermaritzburg 3209, South Africa; 220113325@stu.ukzn.ac.za (M.O.); govenderl3@ukzn.ac.za (L.G.); siwelam@ukzn.ac.za (M.S.); 2 Discipline of Pharmaceutical Sciences, School of Health Sciences, College of Health Sciences, University of KwaZulu-Natal, Durban 4001, South Africa; 221111291@stu.ukzn.ac.za (E.E.); govenderth@ukzn.ac.za (T.G.); 3 Discipline of Chemistry, School of Chemistry and Physics, College of Agriculture, Engineering and Science, University of KwaZulu-Natal, Durban 4000, South Africa; koorbanally@ukzn.ac.za

**Keywords:** asiatic acid, drug release, oleanolic acid, oral administration, solid lipid nanoparticles, solubility

## Abstract

**Objectives:** This study characterised and evaluated the stability, solubility, and in vitro drug release of OA- and AA-loaded SLNs. **Methods:** The OA- and AA-SLNs were formulated using the emulsion solvent evaporation method and characterised based on particle size (PS), polydispersity index (PDI), zeta potential (ZP), and transmission electron microscopy (TEM). Solubility studies were conducted in PBS (pH 1.2 and 6.8) and dH_2_O using HPLC, while in vitro drug release was assessed in simulated intestinal fluid (SIF) (pH 6.8). **Results:** The optimised OA-SLNs (1:1 drug-to-lipid ratio) showed PS, PDI, ZP, and EE% values of 312.9 ± 3.617 nm, 0.157 ± 0.014, −17.0 ± 0.513 mV, and 86.54 ± 1.818%, respectively. The optimised AA-SLNs (1:2 drug-to-lipid: ratio) had a PS of 115.5 ± 0.458 nm, PDI of 0.255 ± 0.007, ZP of −11.9 ± 0.321 mV, and EE% of 76.22 ± 0.436%. The SLNs remained stable for 60 days at 4 °C and room temperature (*p* < 0.05). The solubility study revealed that free OA and AA showed no measurable values in the three solvents. However, OA-SLNs showed the highest solubility in H_2_O (16-fold) followed by PBS at pH 6.8 (10-fold) and pH 1.2 (10-fold). AA-SLNs significantly improved the solubility in PBS at pH 6.8 (88-fold), compared to dH_2_O (6-fold) and PBS at pH 1.2 (26-fold). In vitro drug release studies showed that OA release from the SLNs was significantly increased within 300 min (*p* < 0.05) compared to the free drug. Similarly, AA release from the SLNs was significantly increased within 300 min (*p* < 0.05) compared to free AA. **Conclusions:** These results demonstrate that SLNs enhance OA and AA solubility and drug release, suggesting a promising strategy for improving oral bioavailability and therapeutic efficacy.

## 1. Introduction

The anti-diabetic and anti-hyperlipidaemic properties of bioactive substances, such as terpenoids, flavonoids, alkaloids, proanthocyanidins, and carotenoids, found in medicinal plants, are mediated through the inhibition of α-amylase, glycogen phosphorylase, α-glucosidase, and pancreatic lipase as well as the scavenging of free radicals [1]. Oleanolic acid (OA) is a naturally occurring organic compound found in food and medicinal plants, including olive trees, ginseng, wild sage, legumes, and hawthorn berries [2,3]. The antioxidant properties of OA have been shown by its ability to reduce malondialdehyde (MDA) levels, which is associated with increased free radicals [2]. The potential of this triterpenoid to preserve the activity of pancreatic beta cells to enhance insulin responsiveness makes it a promising anti-diabetic drug [4]. Another triterpene with several biological effects is asiatic acid (AA), which possesses anti-inflammatory, anti-oxidant, and anti-diabetic properties [5,6]. By enhancing insulin sensitivity, AA, found in *Centella*, brown mustard, basil, and daylily, improves blood glucose control [7]. The water solubility of about 40% of commercially available medications is poor, making it challenging to attain controlled absorption from the gastrointestinal tract and adequate bioavailability of the medications [8]. A compound with low aqueous solubility typically absorbs poorly, which lowers its oral bioavailability [9].

**Figure 1:**
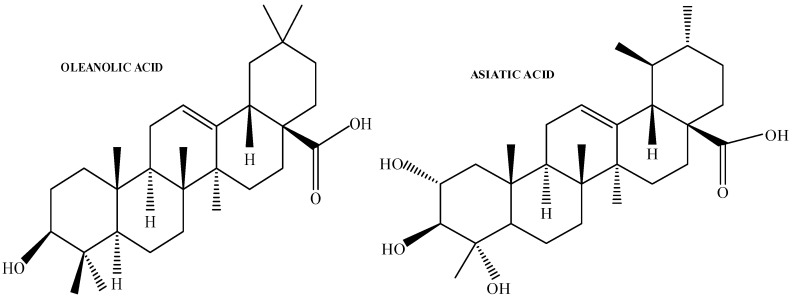
Chemical structures of OA and AA.

Despite the reported biological activity of OA and AA (Figure 1), their poor solubility and low bioavailability have limited their clinical application [10]. According to the biopharmaceutical classification system, OA falls into class IV due to its poor aqueous solubility [11]. Studies indicated that drug delivery technologies that improve the solubility of OA may help with absorption and, consequently, bioavailability [11]. Oral administration of free OA to rats showed poor in vivo absorption and bioavailability (0.7%) [12]. Furthermore, several pharmacokinetic investigations demonstrated that the elimination half-life of OA was comparatively short (<1 h), and the peak plasma concentration of OA after oral administration of doses 300 mg/kg was low. However, the pharmacokinetic profile also suggests tissue distribution found in the heart, liver, kidney, colon, bladder, brain, spleen, lungs, stomach, and testis of animals [11]. Drugs with low solubility and variable absorption have a decreased bioavailability, eventually compromising their effectiveness and safety [13]. Poor drug solubility is a major issue in drug formulation since the drug needs to be in solution for effective administration and absorption [14]. In the literature, several studies have been conducted to improve the solubility, bioavailability, and overall therapeutic efficacy using nanoformulations [14]. Lipid-based nanoparticles such as solid lipid nanoparticles (SLNs) have attracted attention for some 20 years due to their capacity to pass through many physiological barriers [15].

The average particle sizes (PS) of SLN drug delivery systems are below 1000 nm [16]. The two experimental methods commonly used to investigate PS and morphological features are transmission electron microscopy (TEM) and dynamic light scattering (DLS) [17]. The reported benefits of SLNs over other drug delivery methods, such as nanoemulsions and liposomes, are their biocompatibility, use of natural components, large-scale production, low toxicity, biodegradability, sustained drug release profile, and improved drug entrapment capacity [18,19,20]. Furthermore, their physicochemical diversity makes SLNs particularly desirable carriers for oral drug delivery. SLNs have the capacity to increase drug bioavailability by increasing drug solubility and enhancing membrane permeability [21]. SLNs can be prepared using various methods such as solvent emulsion evaporation, membrane contractor, high-pressure, ultrasonication, and hot or cold high-shear homogenisation methods [22]. In the formulation of nanoparticles, polysorbate 80 (C_64_H_124_O_26_), a commonly used hydrophilic nonionic surfactant with low toxicity in food products, cosmetics, and health products, improves drug solubility [23,24]. A previous study showed that polysorbate 80 improved the bioavailability of digoxin (by enhancing absorption) after oral administration in rats [25]. Glyceryl behenate, characterised by long-chain fatty acids, is a lipid used in the formulation of nanoparticles because of its drug entrapment property. The better capacity of glyceryl behenate for drug entrapment is attributed to its complicated structure and less-perfect orientation, which allows for more area for the drug to be loaded [26].

Lipophilic drugs with poor aqueous solubility are encapsulated and effectively delivered using SLNs. During the formulation, the lipid is heated above its melting point for proper drug mixing in the lipid matrix (lipid phase) and then mixed with the aqueous phase (surfactant and water). However, the primary determinants of drug distribution in the lipid matrix include the melting point and polarity of the drug, the properties of the lipid material, and the concentration of the surfactant. The drug entrapment efficiency of SLNs is higher in lipophilic compounds than in polar compounds [27]. A study reported that SLNs formulated using glyceryl behenate had an 8% increase in entrapment efficiency and good zeta potential (ZP) compared to glyceryl monostearate and stearic acid, attributed to the interbedding and improved interaction between molecules provided by the lipid matrix [22]. Enhanced lipids (glycerides) lipophilicity improves solubility and entrapment efficiency of hydrophobic compounds within the SLNs [28]. In addition, the resultant nanoparticles often contain low multiple lattices of lipids having a mixture of mono-, di-, and triglycerides. The less densely packed lattice becomes beneficial as it creates space to facilitate the nanoparticle matrix’s retention of molecules (bioactives).

In vitro drug release testing is a crucial quality-control technique for developing novel treatment formulations regardless of the mode of administration [29]. The release mechanism of a drug from the SLN matrix is determined by the lipid and its composition. The drug can be incorporated either in the matrix or on the surface, and this system can demonstrate flexible release or dual release [30]. Despite the anti-diabetic potential of OA and AA and the technical limitations of their delivery, a scarcity of studies has been conducted to determine whether the two compounds could be delivered in the form of SLNs. Previous studies on OA-loaded SLNs have used film and ultrasound dispersion as well as high-pressure homogenisation methods [31]. A study on the nanoformulation of AA aimed to improve its absorption through intranasal administration (nose-to-brain delivery) [32]. The objectives of the current study were to prepare and characterise OA and AA using the emulsion solvent evaporation method and study the solubility, stability, and in vitro drug release properties of the nanoformulations. By potentially improving the solubility of OA and AA through SLN formulation and investigating the in vitro drug release, this study significantly contributes to the growing area of research on drug delivery strategies for improving the efficacy of plant-derived lipophilic compounds.

## 2. Materials and Methods

### 2.1. Chemicals and Reagents

Isolation of OA (3β-hydroxy-olean-12-en-28-oic acid) was achieved from *Syzygium aromaticum*, and AA and polysorbate 80 were purchased from Sigma-Aldrich Co., Ltd. (St. Louis, MI, USA). Glyceryl behenate was a gift sample from Gattefossé (Saint-Priest Cedex, France). Trehalose dihydrate was purchased from EMD Millipore Corp. (400 Summit Dr. Burlington, MA, USA, an affiliate of Merck KGaA, Darmstadt, Germany). A mill-Q water purification system (Millipore Corp., Burlington, MA, USA) was used to obtain purified milli-Q^®^ water. All chemicals and reagents utilised in this study were procured from reputable commercial suppliers and were of high-quality analytical grade.

### 2.2. Isolation and Characterisation of OA from Cloves

A standard protocol was used to isolate OA from cloves of *Syzygium aromaticum* [(Linnaeus) Merrill & Perry] (Myrtaceae) [33,34]. Cloves were purchased from Sentra Kies Supermarket (Durban, South Africa). The plant material (2 kg) was pulverised and extracted with 5 L of hexane by placing the sample on the mechanical shaker for at least three days. The sample was then sequentially extracted with dichloromethane and ethyl acetate for three days. The ethyl acetate crude extract was subjected to silica gel column chromatography, starting with 100% hexane and continuing with the hexane:ethyl acetate (9:1, 8:2, 7:3) solvent system. Then, 30 mL fractions were collected from each solvent system and analysed using thin-layer chromatography (TLC). Fractions 17–20 (8:2) and fractions 1–23 (7:3) yielded OA. Fractions comparable to the retention factor (R_f_) of standard OA were concentrated using the rotary evaporator at 70 °C. A small amount of chloroform was added to the concentrates and allowed to air dry overnight, and spectroscopic analysis confirmed the structure using ^1^H and ^13^C nuclear magnetic resonance techniques. This approach was chosen to obtain an adequate amount of OA more affordably. The supplier of AA was Sigma-Aldrich Co., Ltd. (St. Louis, MI, USA).

### 2.3. Preparation and Characterisation of OA- and AA-Loaded SLNs

**Figure 2:**
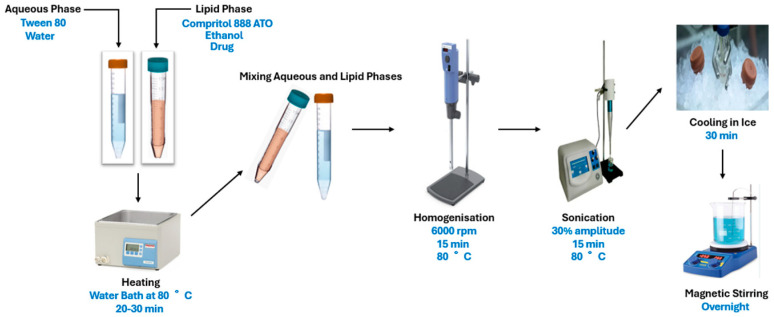
Schematic illustration of the preparation of OA- and AA-loaded SLNs.

The SLNs of OA and AA were prepared using the emulsion solvent evaporation method, consisting of an aqueous and lipid phase [35]. The lipid phase was formulated using glyceryl behenate (10–40 mg), ethanol (3 mL), and the drug (10 mg). To develop the aqueous phase, the surfactant (polysorbate 80; 20–80 mg) was dissolved in 10 mL of distilled water. Both phases, in their liquid states, were incubated separately in a water bath (Scientific) at 80 °C for 20–30 min to produce homogenous phases (Figure 2). Heating was necessary to partially melt the lipid (melting point of 75 °C) and allow the drug to be incorporated into the lipid matrix [18,36]. Ethanol was added to the lipid phase to enhance the solubility of the drug, and the beaker (covered with a foil) and water bath were properly covered to minimise evaporation to quickly enhance the drug’s dissolution. The aqueous phase was added to the lipid phase while inside the water bath. The mixture, while inside the water bath (80 °C), was homogenised using the ultra-homogeniser T18 Ultra Turrax (IKA Labortechnik, Staufen, Germany) at 6000 rpm for 15 min, followed by sonication (Omni Sonic-Ruptor 400 Ultrasonic Homogeniser, Kennesaw, GA, USA) at 30% amplitude for 15 min. The resultant SLNs were quickly cooled in an ice bath for 30 min. The prepared SLNs were stirred overnight to ensure that the formulation was free of organic solvent, which was assumed to be negligible. The final volume was adjusted to 10 mL using distilled water if necessary.

#### 2.3.1. Particle Size, Polydispersity Index, and Zeta Potential Determination

The PS, ZP, and PDI of OA- and AA-loaded SLNs were determined using the dynamic light scattering (DLS) method. In brief, 100 μL of the SLNs of each sample was diluted with 900 μL of distilled water in a glass cuvette cell. Readings were determined in triplicates at 25 °C with a Zetasizer Nano series ZS90 (Malvern Instruments Ltd., Worcestershire, UK) fitted with a 633 nm laser at 173° detection optics. Malvern Panalytical DTS Zetasizer Software v6.02 was used for data collection and analysis.

#### 2.3.2. Transmission Electron Microscopy

The morphology of the SLNs was analysed using TEM (Jeol, JEM-1010, Tokyo, Japan). The SLN samples were properly diluted in distilled water, and a drop was placed on a grid surface, which was allowed to dry at room temperature. Thereafter, using filter paper, the leftover samples were removed. Then, 2% phosphotungstic acid staining agent was added. The samples were allowed to stand for at least 30 s and then visualised (at an accelerating voltage of 75 kV), and the images were captured. The magnifications used were 40,000×, 20,000×, and 8000× for 100, 200, and 500 nm scale, respectively.

#### 2.3.3. HPLC Analysis Method

The calibration curve for OA and AA was determined using a validated HPLC method described by Zhao [37] and Hebbar [38], respectively, with slight modifications. A stock methanolic solution of OA (1000 µg/mL) was prepared, followed by the preparation of various concentrations of 100, 200, 300, 400, 500, 600, and 700 µg/mL and made up to 10 mL with methanol. The HPLC analysis used a C18 column 150 × 4.6 mm (5 µm particle size), mobile phase (acetonitrile: water (95:5)), 1.0 mL/min flow rate at 25 °C, and 20 µL injection volume at 214 nm. For AA, a stock solution of 500 µg/mL was prepared in methanol; after that, concentrations of 10, 20, 30, 40, 50, 60, 70, 80, 90, and 100 µg/mL were prepared by adjusting to 10 mL with methanol. The mobile phase comprises 0.1% orthophosphoric acid:acetonitrile (50:50 *v*/*v*), 10 µL injection volume, 1.0 mL/min flow rate, and read at 214 nm. The calibration curves were plotted by performing a linear regression analysis of the peak area versus OA and AA concentrations.

### 2.4. Entrapment Efficiency (EE%) of OA- and AA-Loaded SLNs

After preparing the SLNs as described in the preparation and characterisation section, the EE% was determined using the ultrafiltration method [39], which involves the separation of the free drug from the encapsulated. For statistical validation, three formulations (F1, F2, F3) for each sample were used to obtain triplicate values. The SLN formulation (2 mL) was pipetted into Amicon^®^ Ultra-4 centrifugal filter tubes (Millipore Corp., Burlington, MA, USA) with a 10 kDa molecular weight cut-off and centrifugated for 30 min at 3000 rpm at 25 °C to obtain the free drug in the supernatant used to determine the concentration of the untrapped drug using the HPLC analysis method (Shimadzu Prominence DGU-20A3, Kyoto, Japan). The values for OA and AA were calculated using the equations and R^2^ values of Y = 6638.1x − 39484 and Y = 4400x − 2741 derived from the calibration curve, respectively. The EE% was calculated using the equation below:

EE%=((Total amount of drug−untrapped drug)/(Total amount of drug))×100

### 2.5. Stability Study of OA- and AA-Loaded SLNs

The stability study examined the effect of storage conditions on the OA- and AA-loaded SLNs. The optimised formulation was physically observed for any phase separation and precipitation, and the PS, PDI, and ZP were measured for 60 days at 4 °C and room temperature (25 °C) to determine the physical stability of the SLNs. Samples of the optimised formulation of OA- and AA-loaded SLNs were collected on days 0, 7, 14, 30, and 60 for PS, ZP, and PDI determination. All values were collected in triplicate.

### 2.6. Solubility Study of OA- and AA-Loaded SLNs

Prior to conducting solubility studies, OA- and AA-loaded SLNs were formulated and freeze-dried using trehalose as a cryoprotectant. Briefly, the SLNs were kept in the biofreezer at −80 °C for 24 h, followed by freeze drying at −42 °C and 212 mT temperature and pressure, respectively, for at least 48 h using a Benchtop Freeze Dryer (SP Scientific VirTis Benchtop Pro Freeze Dryer, Warminster, PA, USA). The solubility of OA- and AA-loaded SLNs was evaluated in phosphate-buffered saline (PBS) pH 1.2 and pH 6.8 as well as distilled water, following a previously reported procedure with a slight modification [40]. For the solubility evaluation, an excess amount of freeze-dried OA- and AA-loaded SLN powder was dispersed in 10 mL of each solvent (PBS pH 1.2, PBS pH 6.8, and distilled water). Similarly, excess pure OA and AA drugs were dispersed in the same solvents as controls. All samples were incubated in a shaking incubator at 37 °C for 72 h. After incubation, 1 mL of each sample was withdrawn, filtered using a 0.22 μm filter, and analysed for drug content using HPLC following the method used for EE%. The solubility values (µg/mL) of OA-loaded SLNs, AA-loaded SLNs, OA, and AA were calculated using the following calibration curve equations: Y = 6638.1x − 39484 for OA and Y = 4400x − 2741 for AA. All experiments were performed in triplicate.

### 2.7. In Vitro Drug Release Profiles of OA- and AA-Loaded SLNs

The drug release properties of the optimised SLNs of OA and AA were studied for 300 min in a SIF (pH 6.8 PBS; 0.1 M) using a dialysis membrane with a molecular weight cut-off (10,000 Da) [41,42]. In brief, OA and AA (10 mg) were dissolved separately in 0.5 mL of dimethyl sulfoxide (DMSO) and completed to 10 mL by distilled water containing sodium dodecyl sulphate (0.3% *w*/*v*). The bare OA and AA solutions (2 mL each) and OA- and AA-loaded SLNs (2 mL each; n = 3) were loaded into separate cellulose dialysis bags and sealed at both ends. The samples were placed in a glass bottle containing 40 mL of SIF and kept in a shaker incubator at a temperature and speed of 37 ± 2 °C and 100 rpm. The sample (2 mL) was drawn from the release medium and immediately supplemented with the same amount (2 mL) of the release medium (PBS pH 6.8) to maintain the sink conditions. The amount of drug released in each 2 mL drawn at 10, 20, 30, 45, 60, 90, 120, 180, 240, and 300 min was analysed by HPLC using the same method stated for EE% evaluation. The experiment was performed in triplicate, and the cumulative release percentage of the drug from the SLNs was estimated using the equation below:

$$\mathrm{Cumulative}\;\mathrm{release}\;\%=\frac{Qt}{Qv}\;\times \;100$$

where Qt is the amount of OA or AA released at time t, and Qv is the total amount of OA or AA previously used in the SLN formulation of OA and AA.

### 2.8. Statistical Analysis

All data are reported as mean ± SD (n = 3). The student’s *t*-test and analysis of variance (ANOVA) were used to determine the differences between two groups and among three or more groups, respectively. The in vitro drug release analysis and graphs were plotted using Microsoft Excel version 2503 (Microsoft Corp., Redmond, Washington, DC, USA) and GraphPad Prism^®^ version 5 (GraphPad Software Inc., San Diego, CA, USA), respectively. The Malvern Panalytical DTS Zetasizer Software version 6.02 was used to collect and analyse PS, PDI, and ZP values. Statistical significance was set at *p* < 0.05.

## 3. Results and Discussion

### 3.1. Characterisation of OA- and AA-Loaded SLN

The formulation components, which consist of lipid (glyceryl behenate), the drug, polysorbate 80, distilled water, and ethanol, are presented in Table 1. Three preliminary optimisations were prepared by varying the drug:lipid ratio, with a fixed lipid surfactant ratio to determine the physicochemical characterisation of OA- and AA-loaded SLNs by measuring the PS, PDI and ZP, and EE% of the formulations as presented in Table 2.

**Figure 3:**
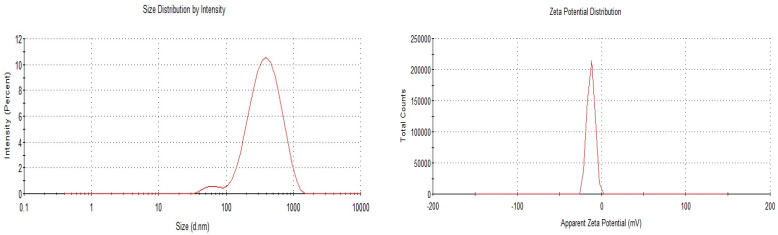
PS and ZP values of optimised OA-loaded SLNs.

**Figure 4:**
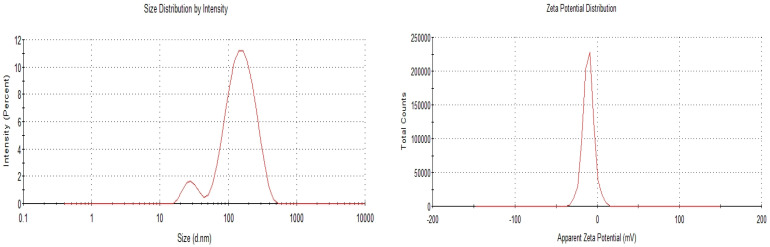
PS and ZP values of optimised AA-loaded SLNs.

Drug:lipid ratios of 1:1 and 1:2 were selected as the optimised SLN formulation for OA and AA, respectively (Table 1). The optimised formulation of each drug was selected based on smaller PS, PDI, and ZP values, which determine the stability of SLNs. Out of the three formulations for OA, the smallest PS was 312.9 nm, and the PDI was 0.157 (<0.3: the recommended value as discussed below). Similarly, the 1:2 ratio was selected as the optimised formulation for AA due to the small PS (115.5 nm) and low PDI (0.255) compared to other formulations. The ZP of both formulations were negatively charged without affecting the stability of the formulations. The PS for OA and AA were below 420 and 200 nm, respectively, within the range for SLNs (40–1000 nm) intended for oral administration (Figure 3 and Figure 4). As reported in previous studies, the PS of OA-loaded SLNs increased with the lipid component. A previous study found that increasing the lipid content by at least 5% was typically related to increased PS, which is attributed to higher PS agglomeration and poor homogenisation efficiency [18]. In this current study, an increase in PS did not affect the EE% of OA-loaded SLNs, which was determined using the equation from the calibration curve described above. Furthermore, considering SLNs formulation and the melting point of glyceryl behenate and the melting point of the drug, a comparison of the EE% of the OA- and AA-loaded SLNs could explain the higher EE% of OA-loaded SLNs compared to AA-SLNs since OA has a lower melting point. In addition, further explanation of the higher EE% of OA-loaded SLNs compared to AA-loaded SLNs could be attributed to the relatively non-polar pentacyclic structure with fewer polar groups of OA, making it more hydrophobic. In contrast, AA has additional hydroxyl groups, increasing its polarity and making it less hydrophobic. The higher hydrophobicity of OA leads to better encapsulation efficiency in hydrophobic environments, resulting in a higher EE% compared to AA. The EE% of a drug increases with its lipophilicity [27]. The low PDI of OA-loaded SLNs (1:1) suggests a narrow PS distribution compared to other ratios, which is essential for consistent drug release and stability of SLNs. Overall, an increase in the lipid content of OA-loaded SLNs increased the PS, PDI, and ZP.

On the other hand, an increase in the lipid content of AA-loaded SLNs reduced the PS of the 1:2 ratio but increased the PS of the 1:4 ratio. In a previous study, at 1% drug and 5% lipid contents, respectively, PS increased but decreased at 10% lipid component due to the presence of higher lipid to entrap more drug, which could result in less entrapment of the drug [43]. The value of ZP, primarily used to characterise the stability of SLNs, represents the potential difference between the electrophoretically mobile particles and the layer around them [44]. The ZP values can affect the pharmacokinetic properties of SLNs and the passage of SLNs through negatively charged cell membranes [45]. The ZP values provide insight into the potential stability and surface charges of nanoformulations [46]. Nanoparticles with a ZP of ±30 mV or more have more significant electrostatic repulsion, and −30 mV or less have less electrostatic repulsion [44]. Hence, it becomes crucial to formulate SLNs with higher ZP to reduce SLN aggregation and improve nanoformulation stability. In this study, the ZP of OA- and AA-loaded SLNs fall within the range of −9 to −17 mV. When electrostatic repulsion overcomes van der Waals forces, SLNs are deemed stable; particle clustering is favoured when electrostatic repulsion is weak (due to low ZP values) [47]. However, because surfactants provide stereochemical stability, some SLN formulations with ZP values below 30 mV maintain their stability over time [48]. A previous study reported that nanoformulation ranging from −18.5 to −41 mV was considered highly negatively charged but with good dispersible stability of the formulation [49]. A study reported that the negativity of ZP could be due to the presence of anionic groups such as carboxyl and phosphate. The negative charge could be affected by the interactions between the different compounds of the SLNs, including the drug. The carboxylic group is present in both OA and AA [50,51]. In addition, a previous study reported the negative charge of ZP of compritol-based SLNs [52]. The SLNs had negative ZP values, contributing to a robust electrostatic repulsive force preventing coalescence or aggregation [53]. However, other parameters determine SLN stability, such as the homogeneous nature of the particles and PDI values, which in our study were within the desired range. About 0.3 or below is acceptable and indicates homogeneous SLN suspensions [44]. In addition, SLNs are characterised by low toxicity and increased surface area, providing an improved drug release profile [30,54].

**Figure 5:**
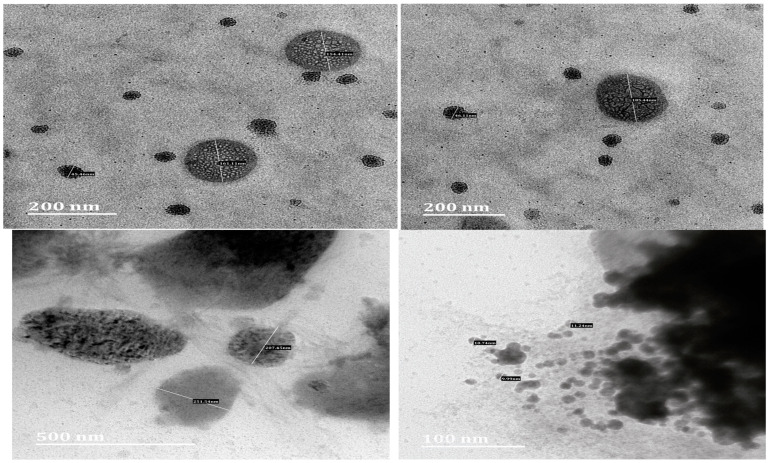
TEM photographs of optimised OA-loaded SLNs (**top**) and AA-loaded SLNs (**bottom**).

The TEM images provide information regarding the PS of the SLNs of OA and AA, and the particles are spherical, as shown in Figure 5. TEM of OA-loaded SLNs showed 161.11 nm and 154.41 nm sizes, while AA-loaded SLNs showed sizes of 207.45 nm and 251.54 nm. The TEM images present nanosized particles without aggregation (larger PS), indicating the stability of the SLNs. These results support the Zetasizer analytical reports that showed the production of SLNs and confirm that the system is within the SLN range of less than 1000 nm [16]. However, the discrepancy between the DLS and TEM sizes could be due to frequently encounters challenging issues associated with the techniques, such as artifacts, actual and formed aggregation, and drying steps, which unavoidably result in non-uniform particle deposition [55,56].

#### Stability of OA- and AA-Loaded SLNs

Good physical stability and resistance to the body’s internal dilution effect define the most promising SLNs [53]. Hence, observing the physical properties of SLNs over time in different conditions is essential in formulating SLNs. The stability of the optimised OA- and AA-loaded SLNs was assessed for two months by measuring the PS, PDI, and ZP (Table 3 and Table 4). The storage conditions for the stability study were 4 °C and room temperature (25 °C). The stability study followed similar protocols and conditions of other studies to provide an understanding of the physical stability of OA- and AA-loaded SLNs. However, for future studies, different parameters can be assessed to provide additional details on the chemical stability of the delivery systems. There were no significant changes in the PS, PDI, and ZP of OA- and AA-loaded SLNs at 4 °C for 60 days (*p* < 0.05). Similarly, there were no significant changes in the PS, PDI, and ZP of OA- and AA-loaded SLNs at room temperature for 60 days. However, there was a significant difference when the PS, PDI, and ZP of the two storage conditions were compared for 60 days (*p* > 0.05). However, at 25 °C, the PDI of OA-loaded SLNs increased from 0.157 ± 0.014 to 0.226 ± 0.014. Similarly, the AA-loaded SLNs showed no significant changes in PS, PDI, and ZP under both storage conditions (4 °C and 25 °C). The results imply that the PS, PDI and ZP for each storage condition for 60 days were significantly stable (*p* < 0.05). A previous study reported no significant changes in PS, PDI and ZP of the formulated SLNs for the delivery of vancomycin at room temperature and 4 °C [39]. Another study reported that AA-loaded SLNs were stable for two months when kept at 4 °C. There was no significant change in the PS over the study period. Under specific conditions, colloidal dispersion can be stabilised at high ZP levels (±30 mV) through electrostatic repulsion, which causes the particles to repel each other and prevent aggregation [44]. As previously reported, the solid phase of the lipid matrix’s SLNs component at room or body temperature is important in the controlled drug release property of drug delivery systems [44]. Our findings are supported by a previous study, which reported that the PS and ZP of SLNs had no significant changes when stored at 4 °C and 25 °C for six months [57]. Another study reported that 4 °C is a favourable storage condition for SLNs, with no drug loss at 20 °C, but an increase in PS was observed at 50 °C [58].

### 3.2. Solubility Study OA- and AA-Loaded SLNs

**Figure 6:**
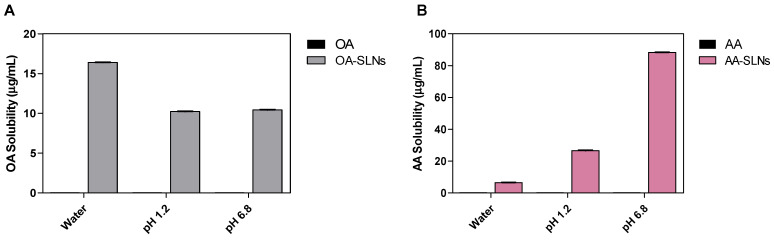
Solubility study of OA-loaded SLNs (**A**) and AA-loaded SLNs (**B**) in water and PBS at pH 1.2 and 6.8. Data are presented as mean ± SD.

The solubility of a drug determines its pharmacokinetic and pharmacodynamic properties, such as absorption, distribution, metabolism, excretion, and receptor binding [59]. Many drug candidates have demonstrated good therapeutic effectiveness in in vitro and in vivo studies. However, their poor water solubility, which impacts their efficacy, bioavailability, and formulation characteristics, is a major cause of their failure in clinical trials [60,61]. The solubility of OA- and AA-loaded SLNs and their bare drugs was evaluated in three solvents: PBS at pH 1.2, PBS at pH 6.8, and distilled water. The results provide significant variations in the solubility of the formulation in solutions with varied pH (Figure 6). The solubility study showed no measurable values for OA and AA bare drug across the three solvents. The OA-SLN solubility data were highest in water (16.40 ± 0.017 µg/mL), followed by PBS at pH 6.8 (10.43 ± 0.032 µg/mL), and PBS at pH 1.2 (10.24 ± 0.006 µg/mL), revealing 16-fold and 10-fold improvements in the solubility of OA in SLNs in water and PBS (pH 1.2 and 6.8), respectively, compared to the free OA. In contrast, AA-loaded SLNs showed the least solubility in water (6.498 ± 0.102 µg/mL) compared to PBS. However, while there was no significant difference in solubility of OA-SLNs in PBS at pH 1.2 and 6.8 (*p* > 0.05), interestingly, AA-loaded SLNs showed significantly higher solubility values at pH 6.8 compared to pH 1.2. In pH 1.2 PBS, the formulation revealed a 26-fold increase in solubility compared to the free drug (26.638 ± 0.198 µg/mL). At pH 6.8, there was an 88-fold improvement in solubility (88.246 ± 0.201 µg/mL).

The solubility of a drug and its formulation is influenced by several factors, including the physicochemical properties of the drug, the composition of the nanoformulation, solvent characteristics, temperature, pH, and PS. Poor water solubility represents a major challenge for orally administered drugs [62], as it significantly impacts the drug’s absorption, distribution, metabolism, and excretion, ultimately affecting bioavailability. SLNs, liposomes, and polymeric microparticles are among the formulation strategies used to enhance the solubility of poorly soluble drugs like OA and AA. Poor aqueous solubility has limited their bioavailability, and SLNs were selected to address this limitation. To validate this, the solubility studies provided data comparing the aqueous solubility of the SLNs to their bare drugs. In this study, OA- and AA-loaded SLNs demonstrated improved solubility compared to their respective free drugs. The improved aqueous solubility supports the rationale behind their formulation and provides insight into the potential of SLNs as a drug delivery system for enhancing the solubility of hydrophobic drugs. These findings are consistent with a previous study, which reported that formulating OA into solid dispersions improved its solubility [41]. Similarly, our results on OA-loaded SLNs are in agreement with a prior study showing that OA-loaded SLNs prepared using the nanoprecipitation method exhibited superior solubility and bioavailability compared to pure OA drug [63].

For AA-loaded SLNs, our solubility results are consistent with earlier findings where AA solubility in water was lower in 1% polysorbate 80 aqueous solution, a surfactant used in SLN formulations [64]. Additionally, previous studies have demonstrated that encapsulating AA in poly lactic-co-glycolic acid nanoparticles enhances its solubility [65]. More interestingly, lipid-based nanoparticles, such as SLNs, may further improve AA solubility and facilitate its delivery to target sites [66]. Moreover, another study reported that AA exhibited higher solubility in saline solution than in water [67], supporting the notion that formulation strategies can significantly enhance its solubility and bioavailability.

### 3.3. In Vitro Drug Release Properties of OA- and AA-Loaded SLNs

The purpose of controlled drug release is to improve efficacy, reduce side effects, and increase compliance [68]. The in vitro drug release properties of OA- and AA-loaded SLNs (1 mg/mL) were conducted in a SIF (pH 6.8) for 300 min using a dialysis bag method. The dialysis bag method is recommended for evaluating the in vitro drug release of SLN drug delivery systems [69]. The drug released from SLNs over 300 min in this study followed previous studies on drug release and provided a preliminary understanding of the release pattern of OA- and AA-loaded SLNs. Future studies might be necessary to provide more details into the release pattern and formulation adjustment over an extended period to optimise the release further. In the first 20 min, there was a burst release and a significant difference (*p* < 0.05) between the OA-loaded SLNs and the free drug. Of note, the SLN preparation method determines the size of the particles as well as the drug’s charging capacity, release capacity, and stability [70]. According to a report, nanoparticles exhibit either a rapid early or biphasic release and a sustained release over several weeks or almost entire drug release over the first few minutes [71]. A wide range of formulations for oral administration with different characteristics can affect drug release from these formulations [72]. A higher or lower release depends on the distribution and entrapment of the drug in the lipid matrix, which can affect a slow or higher release. Drug release patterns can either exhibit a fast release at first, followed by a slow zero- or first-order release of a sustained component or a slow zero- or first-order rate [72]. In addition, the conditions of the release study, including sink or non-sink settings, the release medium, enzyme activity, release time, lipid matrix, sample separation methods, and release by dialysis bag, all affect the drug release kinetics and results [18].

**Figure 7:**
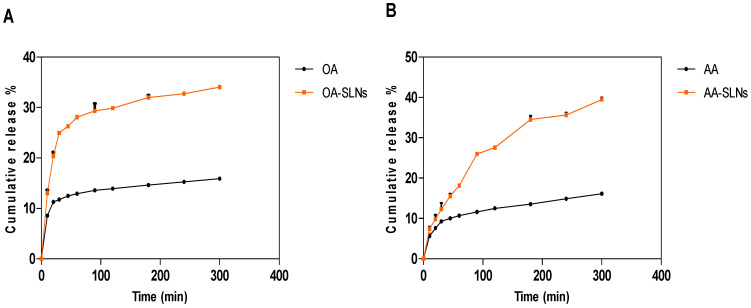
In vitro release profile of (**A**) OA-loaded SLNs and free OA as well as (**B**) AA-loaded SLNs and free AA for 300 min. The cumulative release of OA and AA from the SLNs was significantly higher compared to free OA and AA. Data are presented as mean ± SD. Statistical significance was set at *p* < 0.05.

The release of the drug after 60 min was 12.91% for the free drug and 28.08% for OA-loaded SLNs. The release of the bare drug was maintained between 90 and 120 min (13.56% to 13.91%), but it increased to 14.6% at 180 min before reaching 15.26% at 240 min and 15.88% after 1 h (300 min). Similarly, OA-loaded SLNs maintained a steady but higher release between 90 min and 120 min (29.30% to 29.86%) before reaching 32.73% at 240 min and 34.04% at 300 min. However, there were no significant differences between the release profile of AA-loaded SLNs and its free drug in the first 20 min. A significant difference was observed from 30 min to 300 min. There was a higher release from AA-loaded SLNs compared to the bare drug. AA-loaded SLNs, after 60 min, released 18.16% and 10.68% of free AA. After 300 min, the SLNs of OA and AA showed releases of 34.04% and 39.46%, compared to the free drugs, with releases of 15.88% and 16.13%, respectively (Figure 7). A further comparison between the releases from OA and AA bare drugs showed a significant difference (*p* < 0.05).

The improved drug release from the SLNs could be attributed to the enhanced solubility and small PS of the SLNs. The drug release pattern observed in this study could be attributed to the interactions between the molecules and the lipid matrix. During the formulation of the nanoparticles, the lipid is heated above its melting point to allow for the incorporation of the drug into the lipid matrix. OA has a lower melting point than AA, implying a solid interaction with the lipid, creating a controlled release pattern and higher entrapment than AA-loaded SLNs. A sustained release is attributed to a high entrapment efficiency and to slow diffusion of the drug from the lipid matrix [73]. The behaviour of SLNs is determined by the arrangement and interaction of the crystalline nature of the lipid, stabilising agents, and active ingredients [74]. According to Mishra (2018), smaller particles, due to their large surface area, result in improved drug release compared to larger particles [30]. Another study reported a cumulative release of 34% at 37 °C and more than 90% at 39 °C of lipophilic 5-fluorouracil from SLNs [75]. Our results demonstrate the improved and controlled release of OA- and AA-loaded SLNs compared to their corresponding free drug solutions. The enhanced and sustained release of the SLNs could be attributed to the improved dissolution of the drugs in the lipid matrix and the high surface area of the SLNs, which could be attributed to the drug nanosize range, leading to improved absorption [76].

## 4. Conclusions

This current study contributes to an expanding body of work on the delivery of OA and AA in SLNs by providing physicochemical characterisation and evaluating the physical stability, solubility, and in vitro drug release profiles of the SLNs compared to their bare drug. OA and AA formulations had suitable PS, low PDI, and appropriate ZP, which are critical for formulation stability. The EE% of OA- and AA-loaded SLNs suggests that the SLNs improved the dissolution of the drugs. OA-loaded SLNs exhibited higher encapsulation efficiency than AA-loaded SLNs, likely due to the higher hydrophobicity of OA. The solubility data suggest that SLNs can improve the physicochemical properties of OA and AA, thereby supporting existing data on the ability of SLNs to enhance the solubility of lipophilic drugs. The drug release studies demonstrated a sustained release profile for both compounds from SLNs compared to their bare forms, supporting the improved solubility and, by extension, the improved bioavailability and potential therapeutic benefit of using SLNs for lipophilic drugs. Further studies should explore the in vitro release possibilities for both triterpenes beyond 300 min, and in vivo studies should be performed to evaluate pharmacokinetic and therapeutic outcomes.

## Figures and Tables

**Table 1:** OA- and AA-loaded SLN formulation composition.

Drug:Lipid Ratio	Drug	Lipid	Polysorbate 80	Ethanol	Water
1:1	10 mg	10 mg	20 mg	3 mL	10 mL
1:2	10 mg	20 mg	40 mg	3 mL	10 mL
1:4	10 mg	40 mg	80 mg	3 mL	10 mL

Fixed ratio: Lipid:surfactant (1:2).

**Table 2:** Characterisation of OA- and AA-loaded SLN optimisation studies.

OA:Lipid Ratio	PS (nm)	PDI	ZP (mV)	EE%
1:1	312.9 ± 3.617	0.157 ± 0.014	−17.0 ± 0.513	86.54 ± 1.818
1:2	346.6 ± 1.617	0.217 ± 0.115	−13.2 ± 0.115	87.80 ± 1.024
1:4	412.2 ± 7.002	0.378 ± 0.074	−9.2 ± 0.357	88.93 ± 0.516
**AA:Lipid ratio**	**PS (nm)**	**PDI**	**ZP (mV)**	**EE%**
1:1	162.0 ± 0.681	0.272 ± 0.006	−12.5 ± 0.208	78.96 ± 0.503
1:2	115.5 ± 0.458	0.255 ± 0.007	−11.9 ± 0.321	76.22 ± 0.436
1:4	189.9 ± 1.818	0.259 ± 0.019	−10.2 ± 0.346	79.59 ± 0.214

OA; oleanolic acid, AA; asiatic acid, PS; particle size, PDI; polydispersity index, ZP; zeta potential, EE%; entrapment efficiency.

**Table 3:** Stability of OA-loaded SLNs.

Day(s)	PS (nm) at 4 °C	PDI at 4 °C	ZP (mV) at 4 °C	PS (nm) at RT	PDI at RT	ZP (mV) at RT
0	312.9 ± 3.617	0.157 ± 0.014	−17.0 ± 0.513	312.9 ± 3.617	0.157 ± 0.014	−17.0 ± 0.513
7	333.3 ± 7.328	0.166 ± 0.243	−11.8 ± 0.337	308.6 ± 1.997	0.189 ± 0.100	−12.9 ± 0.302
14	321.5 ± 2.444	0.154 ± 0.026	−9.8 ± 0.100	314.5 ± 1.856	0.191 ± 0.026	−11.1 ± 0.321
30	319.7 ± 1.514	0.146 ± 0.003	−14.4 ± 0.462	311.2 ± 0.551	0.196 ± 0.011	−14.9 ± 0.656
60	320.0 ± 0.800	0.142 ± 0.018	−13.8 ± 0.361	312.5 ± 4.232	0.226 ± 0.014	−15.4 ± 0.200

**Table 4:** Stability of AA-loaded SLNs.

Day(s)	PS (nm) at 4 °C	PDI at 4 °C	ZP (mV) at 4 °C	PS (nm) at RT	PDI at RT	ZP (mV) at RT
0	115.5 ± 0.458	0.255 ± 0.007	−11.9 ± 0.321	115.5 ± 0.458	0.255 ± 0.007	−11.9 ± 0.321
7	116.0 ± 2.313	0.266 ± 0.009	−8.7 ± 0.782	113.3 ± 0.656	0.265 ± 0.005	−9.3 ± 0.387
14	117.3 ± 1.929	0.257 ± 0.010	−7.8 ± 0.391	123.1 ± 4.203	0.277 ± 0.016	−7.6 ± 0.134
30	116.2 ± 2.325	0.259 ± 0.004	−11.8 ± 0.666	114.7 ± 0.404	0.249 ± 0.006	−10.3 ± 0.366
60	119.0 ± 0.265	0.263 ± 0.016	−10.4 ± 0.115	118.1 ± 3.166	0.257 ± 0.050	−8.7 ± 1.220

RT; room temperature, PS; particle size, PDI; polydispersity index, ZP; zeta potential.

## Data Availability

This research article contains most of the data analysed in this study. Any further data requested by interested researchers will be made available upon request.
